# Supplementary material for: Potential Effects of Essential Oil from Plinia cauliflora (Mart.) Kausel on Leishmania: In Vivo, In Vitro, and In Silico Approaches
Source: Microorganisms. 2024 Jan 19;12(1):207. doi: 10.3390/microorganisms12010207 (PMC10819817; doi:10.3390/microorganisms12010207)
Supplement: Supplementary file 1 [file microorganisms-12-00207-s001.zip › microorganisms-2715461-supplementary.pdf]

Article

Potential of Essential Oil from *Plinia cauliflora* (Mart.) Kausel on Leishmania and its complications: *in vivo*, *in vitro*, and *in silico* approaches

Vanderlan N. Holanda[1,*], Thaíse G. S. Brito[1], João R. S. de Oliveira[1], Rebeca X. da Cunha[1], Ana P. S. da Silva[1], Welson V. da Silva[2], Tiago F. S. Araújo[3], Josean F. Tavares[4], Sócrates G. dos Santos[5], Regina C. B. Q. Figueiredo[2] and Vera L. M. Lima[1,*]

Correspondences: Vanderlan N. Holanda (Vanderlan.holanda@ufpe.br) and Vera L. M. Lima (lima.vera.ufpe@gmail.com). Laboratório de Lipídios e Aplicação de Biomoléculas em Doenças Prevalentes e Negligenciadas. Departamento de Bioquímica, Centro de Biociências, Universidade Federal de Pernambuco, Avenida Professor Moraes Rego, 1235, 50670-901 Recife, PE, Brazil.

Supplementary Material

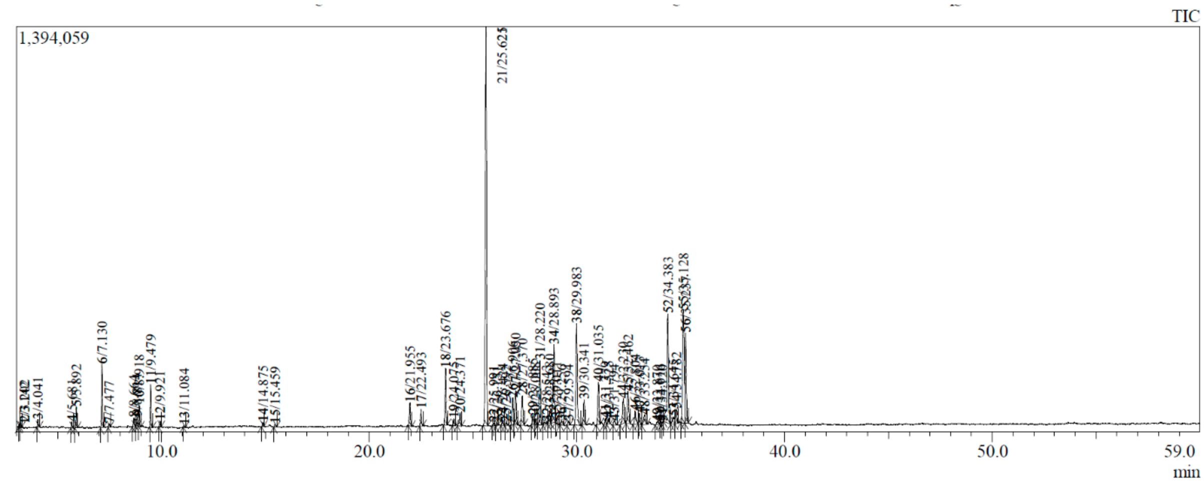

Figure S1. Spectral and chromatographic characteristics of PCEO composition.
